# Inhibition of co-occurring weeds and young sugarcane seedling growth by perennial sugarcane root extract

**DOI:** 10.1038/s41598-024-58082-y

**Published:** 2024-04-01

**Authors:** Xiaoming Wang, Shilong Wang, Jinghuan Zhu, Lei Li, Junjun Ma, Linzhi Zuo, Xiaobo Sun, Bi Chen, Zuli Yang

**Affiliations:** 1 Key Laboratory for the Green and Efficient Production Technology of Sugarcane, Guangxi Science & Technology Normal University, Laibin, China; 2 Ministry of Education Key Laboratory for Ecology of Tropical Islands, Hainan Normal University, Haikou, China; 3 Institute of Analysis and Testing, Beijing Academy of Science and Technology (Beijing Center for Physical & Chemical Analysis), Beijing, China; 4 Laibin Comprehensive Experiment Station of National Sugar Industry Technical System, Laibin Academy of Agricultural Sciences, Laibin, China

**Keywords:** Plant ecology, Plant stress responses, Ecology, Plant sciences

## Abstract

Allelopathy is a process whereby a plant directly or indirectly promotes or inhibits growth of surrounding plants. Perennial sugarcane root extracts from various years significantly inhibited *Bidens pilosa, Digitaria sanguinalis*, sugarcane stem seedlings, and sugarcane tissue-cultured seedlings (*P* < 0.05), with maximum respective allelopathies of − 0.60, − 0.62, − 0.20, and − 0.29. Allelopathy increased with increasing concentrations for the same-year root extract, and inhibitory effects of the neutral, acidic, and alkaline components of perennial sugarcane root extract from different years were significantly stronger than those of the control for sugarcane stem seedlings (*P* < 0.05). The results suggest that allelopathic effects of perennial sugarcane root extract vary yearly, acids, esters and phenols could be a main reason for the allelopathic autotoxicity of sugarcane ratoons and depend on the type and content of allelochemicals present, and that allelopathy is influenced by other environmental factors within the rhizosphere such as the presence of old perennial sugarcane roots. This may be a crucial factor contributing to the decline of perennial sugarcane root health.

## Introduction

Allelopathy is a process by which a plant can directly or indirectly promote or inhibit the growth of surrounding plants, including other individuals of the same species and microorganisms, and occurs via the release of chemical substances into the environment^1,2^. The chemical substances that are responsible for allelopathy are mainly sourced in plant root exudates, aboveground secretions, plant residues or decomposing materials, and extracts^3^. Allelopathy can affect the competitive ability of plant communities within an ecosystem^4,5^. For example, *Stellera chamaejasme* secretes allelochemicals that suppress the germination rate (GR), root and shoot growth, and biomass of co-occurring plants, thus enhancing its own competitive ability^6^. Crops in agricultural systems compete for resources and use root-derived allelochemicals to affect weed germination and growth^7^. For example, rapeseed (*Brassica napus*) can affect the germination and seedling growth of invasive weeds such as redroot pigweed (*Amaranthus retroflexus*) and barnyard grass (*Echinochloa colonum*), and sunflowers (*Helianthus annuus*) suppress the emergence of sunflower broomrape (*Orobanche cumana*) by secreting allelochemicals from their roots^8^. The secretion of allelochemicals by plant roots is the main pathway by which these chemicals enter and accumulate in the soil environment^9^. Allelopathic substances are widely present in various agricultural practices such as perennial cultivation, crop rotation, intercropping, relay cropping, mixed cropping, and fallowing with crop residues, and are also prevalent in the interactions between crops and weeds. Allelopathy can have a significant impact on perennial crops in particular.

The occurrence of sugarcane ratoon decline can be attributed to accumulation of allelopathic autotoxic substances within the soil^10–12^. Research has found that soil extracts in the rhizosphere of both newly planted and perennial sugarcane affect lettuce seedlings via allelopathy, exhibiting a “low concentration promotion, high concentration inhibition” effect, and that the inhibitory effect is stronger in the case of perennial sugarcane than in that of newly planted sugarcane^12^. An aggregate of 54 substances, including acids, phenols, and esters, which may be related to allelopathy, were detected in sugarcane rhizosphere soil^13^. Few previous studies have reported the involvement of allelopathy in sugarcane ratooning, and have mainly focused on changes in the structure, activity, and function of the soil microbial community in the ratoon rhizosphere^14,15^. Currently, no reports elucidating the allelopathy, especially the autotoxic effects, of the chemical components of the sugarcane ratoon root system are available. The rhizosphere environment surrounding sugarcane cultivation is highly complex, mainly as a result of differences in field management and planting years, leading to variations in the composition, quantity, and concentration of the allelochemicals secreted and degraded by sugarcane roots. Separating and analysing the different components of the allelochemicals secreted and degraded by sugarcane roots can help to scientifically clarify the composition of allelochemicals responsible for the decline in ratoon sugarcane growth. In this study, two common malignant weeds, *Bidens pilosa* and *Digitaria sanguinalis*, which coexist in the natural ecological environments of sugarcane fields, were selected alongside sugarcane stem seedlings and sugarcane tissue-cultured seedlings as receptor plants. Different extract concentrations were prepared using sugarcane roots from different years as donor materials and were used to treat receptor seeds (seedlings). The bioassay filter paper method was used to study the associated allelopathy in terms of germination, growth, and biomass accumulation. Thus, we aimed to explore the strength of sugarcane root extract allelopathy and clarify the allelopathic relationships, rules, and characteristics describing the sugarcane root system and the coexisting weeds, sugarcane stem seedlings, and healthy sugarcane seedlings in a field ecosystem. The results of this study are expected to provide both experimental data and a theoretical basis upon which the mechanism of sugarcane root decline can be studied.

## Methods

### Experimental materials

Perennial sugarcane roots of the Guiliu 05/136 variety were selected from first-, second-, and third-year sugarcane plots in the Liangtang “double high sugarcane” land consolidation zone of Xingbin District, Laibin City, Guangxi, China using five-point sampling. The aboveground parts were removed before collection of the underground parts to ensure integrity of the roots. After gently shaking to remove soil and impurities, roots were placed into sample bags and transported to the laboratory for further processing.

*B. pilosa* and *D. sanguinalis* were collected from the National Modern Agriculture Industry Park in Xingbin District, Laibin, Guangxi. Seeds were selected based on uniform maturity and fullness.

Sugarcane stem was collected from newly planted Guiliu 05/136 at the Liangtang “double high sugarcane” land consolidation zone, Xingbin District, Laibin City, Guangxi. Healthy whole stems of plants > 1.2 m in height that were not yet mature and were of uniform quality were selected for processing. Sugarcane stems were cut into segments including one bud, and nodes removed 2.5 cm from the stem were reserved for later use.

Sugarcane tissue-cultured Guiliu 05/136 seedlings of approximately 10 cm height and exhibiting good growth and uniform development were purchased from the Zhanjiang Agricultural Science Research Centre.

We confirm that the use of plants and seeds in the present study complies with international, national and institutional guidelines. The manuscript contains third party material and obtained permissions are available on request by the Publisher.

### Experimental methods

#### Sugarcane root extract preparation

The collected sugarcane roots were carefully brushed with a sterile brush to remove any soil, subjected to air or mechanical drying at temperatures < 45 °C, and cut into small pieces ≤ 2 cm. Samples were then mechanically crushed and passed through a 40-mesh sieve, after which 60 g of sieved sample was removed, placed in a triangular flask, and 1,000 mL of ultrapure water was added. The flask was sealed and tightened, placed in a constant temperature oscillator, and oscillated for 5 d at 25 °C and 100 r/min. The resulting mixture was filtered using a vacuum pump to obtain 60 g/L crude extract concentrations, which comprised sugarcane from different years (J1, J2, and J3) and different root types. Crude extract was then diluted with ultrapure water to obtain 20 and 40 g/L extracts, which were stored at 4 °C for later use. Taking the root extract from the first sugarcane year and mature roots as an example, three samples with concentration gradients of 20, 40, and 60 g/L were prepared and denoted as J1-1, J1-2, and J1-3, respectively^16^. The blank control (CK) was treated with ultrapure water only.

#### Separation of different sugarcane root system components

Concentrated water extracts from the first-, second-, and third-year sugarcane root systems, each with a concentration of 40 g/L, were extracted with ethyl acetate three times at a ratio of 1:1 for 0.5 h to generate a neutral component. The remaining water phase was then adjusted to pH 2 with 3 mol/L HCl, and extracted three times with ethyl acetate at a ratio of 1:1 for 0.5 h to produce the acidic component. The remaining water phase was then adjusted to pH 8 with 3 mol/L NaOH, and extracted three times with ethyl acetate at a ratio of 1:1 for 0.5 h to produce the alkaline component. Taking the different components of the first-year sugarcane root system as an example, the three components were abbreviated as J1-neutral, J1-acidic, and J1-alkaline, respectively. The CK was treated with ultrapure water.

#### Biological activity assay of allelopathy

Uniform and healthy recipient seeds (stems) were selected, immersed in 1% formaldehyde solution for 20 min for disinfection, and rinsed with ultrapure water three times. Sugarcane root extracts from different years were tested at three concentrations (20, 40, and 60 g/L) with three replicates per treatment. The dish filter paper method was used to cultivate *B. pilosa* and *D. sanguinalis* seeds in a programmable artificial climate chamber^17,18^, using ultrapure water to produce the CK. Culture dishes with a 10 cm diameter were washed, dried, and sterilised to produce aseptic culture dishes. Two layers of aseptic qualitative filter paper were laid in each culture dish, and 7 mL of extract at different treatment concentrations was added to the culture bed of each dish. A total of 30 seeds were sown in each culture dish and placed in a programmable artificial climate chamber for germination and cultivation at 35 °C, with 70% humidity and a 10:14 h light:dark photoperiod. Seed germination data were collected and recorded daily. Every other day, an appropriate amount of the corresponding concentration of extract or ultrapure water was added to the culture flask up to the initial water level. Seed germination was assessed according to the standard of embryo roots piercing the seed coat by at least 1 mm. The germination test was terminated on the 10^th^ day, when root length and seedling height were measured using a digital calliper to an accuracy of 0.01 mm and seedling fresh weight was weighed using an analytical balance to an accuracy of 0.0001 g.

Sugarcane stem seeds were cultured using the germination tray filter paper method in a controlled environment chamber, with ultrapure water used for CK. Metal germination trays (50 × 30 × 4 cm length, width, and height, respectively) were washed, dried, and sterilised. Two layers of sterile qualitative filter paper were then placed in each germination tray, and 500 mL of extracted solutions was injected into the tray beds. Twenty sugarcane stem shoots were then placed in each germination tray, covered with two layers of polyethylene film to maintain the humidity, and placed in a programmable artificial climate chamber for germination at 32 °C under 70% humidity and with 10:14 h light:dark photoperiod. Data describing the germination of the stem cuttings were recorded daily, with corresponding extraction solution concentrations or ultrapure water added to the germination tray according to the design. The standard for stem germination was a sugarcane sprout with a length ≥ 1 cm. The germination experiment was completed on day 20, when root length and seedling height were measured using a digital calliper with an accuracy of 0.01 mm. Seedling fresh weight was obtained using an analytical balance with an accuracy of 0.0001 g.

Sugarcane tissue-cultured seedlings were cultivated hydroponically in plant tissue culture bottles in a controlled environment chamber. Ultrapure water was used for the CK instead of extraction solution. Sugarcane tissue-cultured seedlings with a uniform height of approximately 10 cm were selected, and the height, root length, and fresh weight of each tissue culture seedling were measured and recorded. The plant tissue glass culture bottles (approximately 250 mL) were washed, dried, and sterilised before use. No bottle caps were used. Equal amounts of different concentrations of extraction solutions were added to each culture bottle, with the requirement that 80% of the sugarcane roots was in contact with the extraction solution. Five sugarcane tissue-cultured seedlings were placed in each culture bottle, with three bottles constituting one replicate. The bottles were then placed in a controlled environment growth chamber set to 32 °C under 70% humidity and a 10:14 h light:dark photoperiod. Growth was checked daily, and corresponding concentrations of extract solution or ultrapure water were added to the culture bottles according to the design. On day 15, the height, root length, and fresh weight of each tissue culture seedling were measured and recorded. The differences between the measurements before and after cultivation were taken as the growth of the tissue culture seedlings in terms of height, root length, and fresh weight.

#### Biological activity assay of different components of extraction solution

Sugarcane stem seedlings and sugarcane tissue-cultured seedlings were used as receptor samples. Equal amounts of neutral, acidic, and alkaline ethyl acetate extracts were added to culture vessels, with equal amounts of ethyl acetate added to the CK group. After ethyl acetate evaporation, equal and appropriate amounts of ultrapure water were added to each sample, and measurements were made using the same method described in Section "Biological activity assay of allelopathy".

#### GC–MS analysis of the extract

GC–MS analysis was performed on 60 g·L^−1^ root extracts from first-, second-, and third-year sugarcane ratoons, which were named J1-3, J2-3, and J3-3, respectively. Prior to GC–MS analysis, the sugarcane root extracts were pre-processed as follows. A suitable amount of HPLC-grade methanol was added to each root extract. Subsequently, the solutions were evaporated until dry in a rotary evaporator, and the residues were re-dissolved using 5 mL HPLC-grade methanol. Finally, the solutions were filtered through a 0.45-μm organic membrane filter. Chromatography was performed using an Agilent Technologies HP-5MS Ultra Inert capillary column (30 m × 0.250 mm × 0.25 μm). The temperatures were programmed, with an initial temperature of 50 °C, ramp rate of 5 °C/min up to 180 °C (hold time of 10 min), and a ramp rate of 10 °C/min up to 220 °C (hold time of 20 min). The total run time was 60 min. The inlet temperature was 250 °C, and the carrier gas was He, with a flow rate of 1 mL/min. The sample injection quantity was 1 μL. Mass spectrometry was performed in the electron ionization mode, with an electron energy of 70 eV, ion trap temperature of 200 °C, and scan range of 50–1000 amu^19^. The NIST Mass Spectral Match (MSMatch) program was used to match the MS data to the NIST MS library and thus determine the components of the root extracts. The percentage content of each component was determined using the area normalization method. The GC–MS spectrometer (Agilent 7890B/5977A GCMSD) and the peak-analysis software were all purchased from Agilent.

#### Data statistics and analysis

GR was calculated as the percentage of normally germinated seeds to the total number of seeds tested. The germination index (GI) was calculated using ∑(Gt/Dt), where Gt is the number of germinated seeds at t days and Dt is the corresponding germination time. The response index (RI) was calculated according to the method described by Williamson and Richardson^20^, where RI = 1–C/T (when T ≥ C) or RI = T/C–1 (when T < C), C represents the control value, and T represents the treatment value. A positive RI value indicates promotion and a negative RI value indicates inhibition. The larger the absolute RI, the stronger the allelopathy intensity^21^. The synergistic allelopathy effect index (SE) is the arithmetic mean of the allelopathy index RI for all test items under the same treatment for the same receptor, thus reflecting the strength of the allelopathic effect. The sensitivity index (SI) is calculated in the same way as the SE and can comprehensively evaluate the strength of allelopathy from different donors, while also evaluating the sensitivity of a receptor to allelopathy^22^. Overall allelopathy, which is calculated separately according to the donor or receptor, is the comprehensive SI.

$$ {\text{SE }} = {\text{ SI }} = \, \left( {{\text{RI}}_{{\text{project 1}}} + {\text{ RI}}_{{\text{project 2}}} + {\text{ RI}}_{{\text{project X}}} } \right) \, /{\text{ X}} $$

Comprehensive SI = Σ (mean SI values for each treatment for all receptors) or Σ (mean SI for each treatment for all donors).

To compare the similarity of compounds in the root extracts of perennial sugarcane from different years, the similarity coefficient formula for S^23^: S = d/(a + b + c + d) × 100% was used, where a, b, and c represent the number of unique components with a relative content > 1% in J1-3, J2-3, and J3-3, respectively, and d represents the number of common components with a relative content > 1%.

### Data processing

Data was statistically analysed using a WPS Office 2016 XLSX worksheet, significant differences were determined Minitab 16, figures were generated using Origin 2018, and analysis of variance was conducted using SPSS 22.0 Experimental data are presented as the mean ± standard deviation.

## Results and discussion

### Effects of root extracts on *B. pilosa* seed germination and seedling growth

As shown in Table 1, the addition of different root extract concentrations from perennial sugarcane in different years resulted in varying degrees of inhibition of the GR, GI, vigour index, embryonic root length, embryonic shoot length, and fresh weight of *B. pilosa*. Except for the GR and embryonic shoot length under treatment J3-1 and the GI under treatment J2-1, which did not differ significantly from the control, all other treatments led to significant allelopathic inhibition (*P* < 0.05). Previous studies have shown that extracts from three *Chenopodium quinoa* organs^24^, *Artemisia selengensis* litter^25^, and different parts of sorghum straw^26^ inhibit germination of weed seeds, which is similar to the results of this study. The allelopathy index indicated that, of the tested parameters, the vigour index was most sensitive to allelopathic inhibition followed by GI, GR, and embryonic root length in *B. pilosa* seeds, and that the allelopathic inhibition of seedling fresh weight and embryonic shoot length was relatively low. Treatment J1-3 showed the strongest allelopathic inhibition effect in terms of viability with an allelopathy index of − 0.89, which was significantly stronger than that of all other treatments except J1-2 and J2-3 (*P* < 0.05). The strongest allelopathic inhibition for GI was observed in J1-3 (allelopathy index of − 0.81), which was significantly stronger than those in other treatments (*P* < 0.05). Allelopathic inhibition of GR was strongest in J1-3 with an allelopathy index of − 0.71, which was significantly stronger than those of other treatments (*P* < 0.05). The strongest allelopathic inhibition effect on embryonic root length was observed in J2-3 with an allelopathic inhibition index of − 0.49, which was significantly stronger than those of all other treatments except J1-3 and J3-3 (*P* < 0.05). The strongest allelopathic inhibition effect on fresh seedling weight was observed in J1-3 with an allelopathy index of − 0.44, which displayed significantly stronger inhibition than any other treatment except J1-2 (*P* < 0.05). J2-3 exhibited the strongest allelopathic inhibition effect in terms of the shoot length of germinated seeds, with an allelopathy index of − 0.31; this was significantly stronger than that of all other treatments (*P* < 0.05). Compared to CK, J1-3 showed significant decreases in terms of vigour (by 89.20%), germination (by 80.70%), GR (by 70.55%), and fresh weight (by 44.06%). Similarly, J2-3 showed a significant reduction of 48.85% in root length and of 31.58% in shoot length compared to CK. Based on the allelopathic synthesis effect index, the allelopathic inhibition effects decreased from strongest to weakest in the following order: J1-3 > J2-3 > J1-2 > J2-2 > J3-3 > J3-2 > J1-1 > J2-1 = J3-1. J1-3 exhibited the strongest allelopathic comprehensive inhibition effect of − 0.60, and J2-1 and J3-1 showed weak allelopathic comprehensive inhibition effects of − 0.16. In terms of overall allelopathy, the overall allelopathic inhibition effects of all treatments were significantly stronger than that of CK (*P* < 0.05). J1-3 displayed a significantly stronger overall allelopathic inhibition effect than any other treatment (*P* < 0.05), and J2-3 exhibited a significantly stronger overall allelopathic inhibition effect than other treatments except J1-3 and J1-2 (*P* < 0.05).

**Table 1:** Effects of extracts on *Bidens pilosa* germination and seedling growth, and allelopathy evaluation.

Treat-ment	Test item						Allelopathic RI						Allelopathic synthesis effect SE
	Germination rate (%)	Germination index	Vitality index	Radicle length (mm)	Shoot length (mm)	Seedling fresh weight (mg)	Germination rate	Germination index	Vitality index	Radicle length	Shoot length	Seedling fresh Weight	
CK	56.7 ± 5.8^a^	1.14 ± 0.12^a^	9.35 ± 1.06^a^	10.4 ± 0.1^a^	19.0 ± 1.1^a^	8.17 ± 0.22^a^	0^a^	0^a^	0^a^	0^a^	0^a^	0^a^	0^a^
J1-1	36.7 ± 3.3d^ef^	0.85 ± 0.11^bc^	5.92 ± 0.36^ cd^	7.50 ± 0.38^d^	16.2 ± 0.3^d^	6.84 ± 0.59^ cd^	− 0.35^ cd^	− 0.26^b^	− 0.37^c^	− 0.28^d^	− 0.15^c^	− 0.16^ cd^	− 0.26^c^
J1-2	33.3 ± 0.0^ef^	0.43 ± 0.06f.	2.14 ± 0.27^ fg^	6.24 ± 0.64^ef^	14.9 ± 0.1^ef^	5.01 ± 0.13^ef^	− 0.41^ cd^	− 0.63^de^	− 0.77^ef^	− 0.40^ef^	− 0.21^d^	− 0.39^ef^	− 0.47^ef^
J1-3	16.7 ± 0.0^ g^	0.22 ± 0.02^ g^	1.01 ± 0.15^ g^	5.43 ± 0.81^ g^	13.7 ± 0.7^gh^	4.57 ± 0.28f.	− 0.71^e^	− 0.81f.	− 0.89f.	− 0.48^ g^	− 0.28^e^	− 0.44f.	− 0.60^ g^
J2-1	46.7 ± 3.3^bc^	1.01 ± 0.14^ab^	7.33 ± 1.19^b^	8.92 ± 0.14^bc^	15.1 ± 0.1^e^	7.50 ± 0.15^b^	− 0.18^b^	− 0.12^ab^	− 0.22^b^	− 0.14^bc^	− 0.20^d^	− 0.08^b^	− 0.16^b^
J2-2	34.4 ± 1.9^ef^	0.44 ± 0.11^ef^	2.69 ± 0.65f.	6.66 ± 0.35^e^	14.3 ± 0.3^ fg^	6.35 ± 0.41^d^	− 0.39^ cd^	− 0.61^de^	− 0.71^e^	− 0.36^e^	− 0.25^e^	− 0.22^d^	− 0.42^e^
J2-3	30.0 ± 5.8f.	0.41 ± 0.12^ fg^	2.10 ± 0.48^ fg^	5.32 ± 0.24^ g^	13.0 ± 0.6^ h^	5.15 ± 0.39^e^	− 0.47^d^	− 0.64^e^	− 0.78^ef^	− 0.49^ g^	− 0.31f.	− 0.37^e^	− 0.51f.
J3-1	52.2 ± 6.9^ab^	0.91 ± 0.12^b^	6.79 ± 1.06^bc^	9.44 ± 0.32^b^	18.4 ± 0.2^ab^	7.44 ± 0.25^b^	− 0.16^b^	− 0.25^b^	− 0.31^bc^	− 0.09^b^	− 0.03^a^	− 0.09^b^	− 0.16^b^
J3-2	44.4 ± 6.9^bcd^	0.70 ± 0.16^ cd^	4.91 ± 1.14^de^	8.20 ± 0.59^ cd^	17.6 ± 0.5^bc^	7.07 ± 0.42^bc^	− 0.29^bc^	− 0.42^c^	− 0.50^d^	− 0.21^ cd^	− 0.07^b^	− 0.13^bc^	− 0.27^c^
J3-3	41.1 ± 6.9^cde^	0.64 ± 0.13^de^	4.26 ± 0.71^e^	5.85 ± 0.43^ fg^	16.7 ± 0.1^ cd^	6.74 ± 0.35^ cd^	− 0.34^c^	− 0.47^ cd^	− 0.57^d^	− 0.44^ fg^	− 0.12^c^	− 0.18^ cd^	− 0.35^d^

Data are the mean ± standard deviation of three replicates. Different lowercase letters in each column represent significant differences at the 0.05 level.

*CK* blank control, *RI* response index, *SE* synergistic allelopathy effect index.

Overall, the phytotoxic effect of the test items increased gradually and the phytotoxic inhibition effect was enhanced with an increase in the concentration of root solution from the same year; i.e., phytotoxic inhibition decreased in the following order: 60 g/L > 40 g/L > 20 g/L. Under the same root solution concentration, increasing the host root age led to gradual weakening in the phytotoxic inhibition effect, which decreased in the following order: J1 > J2 > J3.

### Effects of root extracts on *D. sanguinalis* seed germination and seedling growth

As shown in Table 2, different concentrations of perennial ryegrass root extracts from different years resulted in varying degrees of reduction in GR, GI, vigour index, embryonic root length, embryonic shoot length, and fresh weight of *D. sanguinalis,* a weed that is commonly associated with sugarcane. With the exception of J3-1, for which no significant difference in GI was observed compared to the control, all treatments exhibited significant allelopathic inhibition (*P* < 0.05). The allelopathy index indicated that the vitality index was most sensitive to allelopathic inhibition, followed by GI, GR, and embryonic root length, and relatively minimal effects were observed in terms of embryonic shoot length and seedling fresh weight. J1-3 exhibited the strongest growth-inhibiting effect in terms of the vitality index, with a phytotoxicity index of − 0.93, which was significantly stronger than that of all other treatments except J1-2 (*P* < 0.05). J1-3 exhibited the strongest allelopathic inhibition effect for germination with an allelopathic index of − 0.89, which significantly surpassed that of all other treatments except J1-2 (*P* < 0.05). GR analysis indicated the most potent allelopathic inhibition effect under J1-3 with an allelopathic index of − 0.78, which significantly surpassed that of all other treatments except J1-2 (*P* < 0.05). Embryonic root length analysis revealed that J3-3 exhibited the strongest allelopathic inhibition effect with an allelopathic index of − 0.69, significantly surpassing that of all other treatments except J2-3 (*P* < 0.05). J2-3 exhibited the strongest allelopathic inhibition effect on embryonic shoot elongation, with an allelopathy index of − 0.44; this was significantly more pronounced than that observed under all other treatments except J2-2 (*P* < 0.05). J2-3 displayed the strongest allelopathic inhibition effect on seedling fresh weight with an allelopathy index of − 0.40, which was significantly more pronounced than that of any other treatment (*P* < 0.05). Compared to CK, J1-3 resulted in a 92.75% decrease in the vigour index, a 89.26% decrease in GI, and a 78.39% decrease in GR. Compared to CK, J3-3 led to a 68.67% reduction in embryonic root length, and J2-3 caused a 44.49% decrease in embryonic shoot length and a 39.68% reduction in seedling fresh weight and led to reductions in embryonic shoot length by 44.49% and in seedling fresh weight by 39.68%. The allelopathic inhibition effects indicated by the allelopathy index decreased from the strongest to the weakest in the following order: J1-3 > J2-3 > J1-2 > J3-3 > J2-2 > J1-1 > J2-1 > J3-2 > J3-1. J1-3 exhibited the strongest allelopathic inhibition effect with an allelopathy index of − 0.62, and J3-1 showed the weakest allelopathic inhibition effect with an allelopathy index of − 0.17. In terms of overall allelopathy, significantly strong allelopathic inhibition was noted for all treatments compared to CK (*P* < 0.05). J1-3 exhibited a significantly stronger overall allelopathic inhibition effect than that of all other treatments except J2-3 (*P* < 0.05). Similarly, J2-3 displayed a significantly stronger overall allelopathic inhibition effect than all other treatments except J1-3 and J1-2 (*P* < 0.05).

**Table 2:** Effects of extracts on *Digitaria sanguinalis* seed germination and seedling growth, and allelopathy evaluation.

Treatment	Test item						Allelopathic RI						Allelopathic synthesis effect SE
	Germination rate (%)	Germination index	Vitality index	Radicle length (mm)	Shoot length (mm)	Seedling fresh weight (mg)	Germination rate	Germination index	Vitality index	Radicle length	Shoot length	Seedling fresh weight	
CK	72.2 ± 5.1^a^	2.42 ± 0.36^a^	18.2 ± 3.3^a^	22.6 ± 1.2^a^	22.7 ± 0.9^a^	7.51 ± 0.24^a^	0^a^	0^a^	0^a^	0^a^	0^a^	0^a^	0^a^
J1-1	31.1 ± 10.2f.	0.94 ± 0.48^de^	5.66 ± 3.03^ef^	10.6 ± 0.2^c^	19.7 ± 0.2^c^	5.90 ± 0.35^ cd^	− 0.57f.	− 0.61^d^	− 0.69^de^	− 0.53^c^	− 0.13^c^	− 0.21^ cd^	− 0.46^d^
J1-2	18.9 ± 6.9^ g^	0.58 ± 0.37^ef^	3.28 ± 2.13^ fg^	10.3 ± 0.4^ cd^	18.5 ± 0.3^d^	5.70 ± 0.10^cde^	− 0.74^ g^	− 0.76^de^	− 0.82^ef^	− 0.54^c^	− 0.19^d^	− 0.24^cde^	− 0.55^ef^
J1-3	15.6 ± 5.1^ g^	0.26 ± 0.06f.	1.32 ± 0.33^ g^	9.34 ± 0.19^e^	17.7 ± 0.2^d^	5.03 ± 0.25f.	− 0.78^ g^	− 0.89^e^	− 0.93f.	− 0.59^d^	− 0.22^d^	− 0.33^ fg^	− 0.62^ g^
J2-1	56.7 ± 3.3^b^	1.74 ± 0.06^bc^	9.65 ± 0.40^ cd^	9.45 ± 0.23^de^	13.5 ± 0.8^e^	5.55 ± 0.17^de^	− 0.22^b^	− 0.28^bc^	− 0.47^c^	− 0.58^d^	− 0.40^e^	− 0.26^de^	− 0.37^c^
J2-2	46.7 ± 3.3^ cd^	1.38 ± 0.24^ cd^	6.94 ± 1.32^de^	8.27 ± 0.25f.	13.0 ± 0.6^e^	5.01 ± 0.10f.	− 0.35^ cd^	− 0.43^c^	− 0.62^d^	− 0.63^e^	− 0.43^ef^	− 0.33^ g^	− 0.47^d^
J2-3	37.8 ± 1.9^ef^	0.88 ± 0.09^e^	3.98 ± 0.34^efg^	7.25 ± 0.08^ g^	12.6 ± 0.2^e^	4.53 ± 0.35^ g^	− 0.48^ef^	− 0.64^d^	− 0.78^e^	− 0.68f.	− 0.44f.	− 0.40^ h^	− 0.57^ fg^
J3-1	53.3 ± 3.3^bc^	2.02 ± 0.32^ab^	13.6 ± 2.4^b^	17.8 ± 0.8^b^	21.6 ± 0.4^b^	6.73 ± 0.22^b^	− 0.26^bc^	− 0.17^ab^	− 0.25^b^	− 0.21^b^	− 0.05^b^	− 0.10^b^	− 0.17^b^
J3-2	44.4 ± 3.8^de^	1.77 ± 0.11^bc^	10.8 ± 0.4^bc^	10.6 ± 0.3^c^	18.5 ± 0.6^d^	6.13 ± 0.48^c^	− 0.38^de^	− 0.27^bc^	− 0.41^c^	− 0.53^c^	− 0.18^d^	− 0.18^c^	− 0.33^c^
J3-3	35.6 ± 1.9f.	0.93 ± 0.16^e^	5.04 ± 0.87^ef^	7.08 ± 0.81^ g^	20.4 ± 0.8^c^	5.44 ± 0.02^ef^	− 0.51f.	− 0.62^d^	− 0.72^de^	− 0.69f.	− 0.10^c^	− 0.28^ef^	− 0.49^de^

Data are the mean ± standard deviation of three replicates. Different lowercase letters in each column represent significant differences at the 0.05 level.

*CK* blank control, *RI* response index, *SE* synergistic allelopathy effect index.

Overall, the phytotoxicity effect in the tested items increased as the concentration of extract from the same year increased (except J3), indicating a pattern of 60 g/L treatment > 40 g/L treatment > 20 g/L treatment for phytotoxicity inhibition. Existing research indicates that water extracts of fallen leaves from *Pinus massoniana* Lamb. at different mass concentrations^27^ and flower extracts from *Tamarix chinensis* Lour^28^ inhibit ryegrass. The overall trend of allelopathic inhibition for sugarcane root extracts with the same concentration tended to gradually decrease as the perennial root age increased, with allelopathic inhibition decreasing in the following order: J1 > J2 > J3. This may have been related to the types and contents of allelochemicals in the roots of perennial plants of different ages, which is consistent with the research results of Bao et al.^29^.

### Effects of root extracts on germination and seedling growth of sugarcane stem

Table 3 reveals that, compared to the CK, different concentrations of root extracts from sugarcane of various ages resulted in varying degrees of reduced GR, GI, root length, seedling height, and fresh weight of sugarcane stem seedlings. All concentration treatments led to significant allelopathic inhibition (*P* < 0.05). Yanxin et al.^30^ found that root extract from *Panax ginseng* had a significant effect on *P. ginseng* seedling growth and physiology, inhibiting their survival, which is consistent with the results of this study. This phenomenon may be due to changes in the physical and chemical properties of the soil and enzyme activity resulting from the presence of the extract. The allelopathy index indicated that, of the items tested, the fresh weight of sugarcane seedlings was the most sensitive to allelopathic inhibition, followed by seedling height and root length, and that GR and GI were less affected. The allelopathic inhibition effect on fresh weight of seedlings was strongest under J2-3, with an allelopathy index of − 0.25, which was significantly stronger than the inhibitory effects observed under J3-1 and J1-1, which were 2.50- and 1.56-times lower, respectively (*P* < 0.05). In terms of seedling height growth, the strongest allelopathic inhibition effect was observed in J2-3, with an allelopathic inhibition index of − 0.33. This effect was significantly stronger than those observed in J1-1, J3-1, J1-2, J2-1, and J3-2 (*P* < 0.05), which were 1.83, 1.83, 1.65, 1.43, and 1.43-times weaker, respectively. In terms of the effect of comprehensive allelopathy on root length, the strongest inhibitory effect was observed in J2-3 with an allelopathy index of − 0.21, which was significantly stronger than that of all other treatments except J3-3 (*P* < 0.05). The allelopathic inhibition effect on GR was the strongest in J2-3 with an allelopathy index of − 0.18, which was significantly stronger than those observed in J1-1, J1-2, and J3-1 (*P* < 0.05). The allelopathic inhibition effect on GI was the strongest in J2-3 with an allelopathy index of − 0.15, which was significantly stronger than those observed in J1-1, J3-1, and J3-2 (*P* < 0.05). Compared to CK, J2-3 resulted in decreases of 24.57% in seedling fresh weight, 20.11% in seedling height, 20.52% in root length, 15.22% in GI, and 17.58% in GR. Based on the comprehensive chemically induced suppression index, the strength of the chemically induced suppression decreased from strongest to weakest in the following order: J2-3 > J3-3 > J1-3 = J2-2 > J2-1 = J3-2 > J1-2 > J3-1 > J1-1. The strongest comprehensive chemically induced suppression was exhibited in J2-3 with an index of − 0.20, and the weakest was exhibited in J1-1 with an index of − 0.10. The suppression effects of J2-3, J3-3, J1-3, and J2-2 were significantly stronger than those of J3-1 and J1-1 (*P* < 0.05).

**Table 3:** Effects of extracts on the germination and seedling growth of sugarcane stems, and allelopathy evaluation.

Treatment	Test item					Allelopathic RI					Allelopathic synthesis effect SE
	Germination rate (%)	Germination index	Root length (cm)	Seedling height (cm)	Seedling fresh weight (g)	Germination rate	Germination index	Root length	Seedling height	Seedling fresh weight	
CK	95.0 ± 5.0^a^	13.8 ± 0.5^a^	9.16 ± 0.14^a^	17.4 ± 0.3^a^	2.93 ± 0.08^a^	0^a^	0^a^	0^a^	0^a^	0^a^	0^a^
J1-1	88.3 ± 2.9^b^	12.8 ± 0.5^b^	8.19 ± 0.21^b^	15.8 ± 0.4^b^	2.46 ± 0.14^b^	− 0.07^b^	− 0.08^b^	− 0.11^b^	− 0.09^b^	− 0.16^bc^	− 0.10^b^
J1-2	86.7 ± 2.9^bc^	12.3 ± 0.6^bc^	7.91 ± 0.24^bc^	15.3 ± 0.5^bc^	2.35 ± 0.07^ cd^	− 0.09^bc^	− 0.11^bc^	− 0.14^bc^	− 0.12^bc^	− 0.20^ cd^	− 0.13^bc^
J1-3	81.7 ± 2.9^ cd^	12.0 ± 0.7^bc^	7.84 ± 0.22^bc^	14.3 ± 0.5^de^	2.30 ± 0.17^ cd^	− 0.14^ cd^	− 0.13^bc^	− 0.14^bc^	− 0.18^de^	− 0.21^ cd^	− 0.16^ cd^
J2-1	81.7 ± 2.9^ cd^	11.9 ± 0.3^bc^	7.96 ± 0.36^bc^	15.8 ± 0.4^b^	2.35 ± 0.15^ cd^	− 0.14^ cd^	− 0.13^bc^	− 0.13^bc^	− 0.09^b^	− 0.20^ cd^	− 0.14^bc^
J2-2	80.0 ± 0.0^d^	12.0 ± 0.3^bc^	7.76 ± 0.33^bc^	15.3 ± 0.6^bc^	2.23 ± 0.12^ cd^	− 0.16^d^	− 0.13^bc^	− 0.15^bc^	− 0.12^bc^	− 0.24^ cd^	− 0.16^ cd^
J2-3	78.3 ± 5.8^d^	11.7 ± 0.5^c^	7.28 ± 0.40^d^	13.9 ± 0.8^e^	2.21 ± 0.14^d^	− 0.18^d^	− 0.15^c^	− 0.21^d^	− 0.20^e^	− 0.25^d^	− 0.20^d^
J3-1	86.7 ± 2.9^bc^	12.8 ± 0.4^b^	7.85 ± 0.26^bc^	15.1 ± 0.4^bcd^	2.64 ± 0.10^b^	− 0.09^bc^	− 0.08^b^	− 0.14^bc^	− 0.14^bcd^	− 0.10^b^	− 0.11^b^
J3-2	83.3 ± 2.9^bcd^	12.6 ± 0.4^b^	7.88 ± 0.28^bc^	15.0 ± 0.5^bcd^	2.34 ± 0.19^ cd^	− 0.12^bcd^	− 0.09^b^	− 0.14^bc^	− 0.14^bcd^	− 0.20^ cd^	− 0.14^bc^
J3-3	78.3 ± 2.9^d^	12.0 ± 0.5^bc^	7.50 ± 0.26^ cd^	14.5 ± 0.6^cde^	2.35 ± 0.18^ cd^	− 0.18^d^	− 0.13^bc^	− 0.18^ cd^	− 0.17^cde^	− 0.20^ cd^	− 0.17^ cd^

Data are the mean ± standard deviation of three replicates. Different lowercase letters in each column represent significant differences at the 0.05 level.

*CK* blank control, *RI* response index, *SE* synergistic allelopathy effect index.

Overall, except for J2-1 and J2-2 in terms of GI and J3-2 and J3-3 in terms of seedling fresh weight, the allelopathy of the other test items gradually increased as the concentration of extract from the same year increased, exhibiting allelopathic inhibition that decreased in the following order: 60 g/L > 40 g/L > 20 g/L. Under the same root extract concentration, allelopathic inhibition showed an overall trend of first increasing and then decreasing as the perennial age increased, with allelopathic inhibition decreasing in the following order: J2 > J1 > J3.

### Effects of soaking solution on growth of sugarcane tissue-cultured seedlings

Table 4 indicates that, compared to CK, the different concentrations of root extracts from sugarcane of different ages significantly inhibited shoot height, root length, and fresh weight of sugarcane plantlets in tissue culture, and all concentrations showed a significant allelopathic inhibitory effect on sugarcane plantlet growth (*P* < 0.05). The allelopathy index revealed that shoot growth and root length were more sensitive to allelopathic inhibition than fresh weight growth. In terms of seedling height, the strongest allelopathic inhibition effect was observed in J2-3 with an allelopathy index of − 0.33, which was significantly stronger than those observed in J1-1, J3-1, J1-2, J2-1, and J3-2 (*P* < 0.05) in which the effects were 1.83, 1.83, 1.65, 1.43, and 1.43-times weaker, respectively. The strongest allelopathic inhibition in terms of root length growth was observed in J2-3 with an allelopathy index of − 0.33, which was significantly stronger than that in J3-1, J2-1, J3-2, J1-1, and J1-2 (2.54 times, 1.74, 1.74, 1.65, and 1.50-times stronger, respectively; *P* < 0.05) J2-3 led to the strongest allelopathic inhibition effect on fresh seedling growth with an allelopathy index of − 0.21, which was significantly stronger than the effects observed under treatments J1-1, J2-1, and J3-1, which were 1.91, 1.75, and 1.75-times lower (*P* < 0.05), respectively. Compared to CK, J2-3 resulted in 32.77%, 33.02%, and 21.26% decreases in plant height, root length, and fresh weight, respectively. The comprehensive allelopathy index indicated that allelopathic inhibition decreased from strongest to weakest in the following order: J2-3 > J1-3 > J2-2 > J3-3 > J1-2 > J3-2 > J2-1 > J1-1 > J3-1. The strongest allelopathic inhibitory effect was observed in J2-3 with a comprehensive allelopathy index of − 0.29, and the weakest was in J3-1 with a comprehensive allelopathy index of − 0.14. Inhibitory effects were significantly stronger in J2-3 and J1-3 than in J3-1, J1-1, J2-1, J3-2, and J1-2 (*P* < 0.05).

**Table 4:** Effect of extracts on the growth of sugarcane tissue-cultured seedlings and allelopathy evaluation.

Treatment	Test item			Allelopathic RI			Allelopathic synthesis effect SE
	Root length growth (cm)	Seedling height growth (cm)	Seedling fresh weight growth (g)	Root length growth	Seedling height growth	Seedling fresh weight growth	
CK	3.21 ± 0.36^a^	23.5 ± 2.0^a^	2.07 ± 0.12^a^	0^a^	0^a^	0^a^	0^a^
J1-1	2.57 ± 0.21^bc^	19.3 ± 1.0^b^	1.83 ± 0.10^b^	− 0.20^bcd^	− 0.18^b^	− 0.11^b^	− 0.16^bc^
J1-2	2.51 ± 0.22^bc^	18.8 ± 1.5^bc^	1.72 ± 0.11^bc^	− 0.22^ cd^	− 0.20^c^	− 0.17^bc^	− 0.20^ cd^
J1-3	2.34 ± 0.24^ cd^	17.0 ± 1.1^cde^	1.67 ± 0.10^bc^	− 0.28^de^	− 0.27^de^	− 0.19^bc^	− 0.25^ef^
J2-1	2.60 ± 0.10^bc^	18.0 ± 0.6^bcd^	1.82 ± 0.12^b^	− 0.19^bc^	− 0.23^bcd^	− 0.12^b^	− 0.18^bc^
J2-2	2.41 ± 0.10^ cd^	16.7 ± 0.8^de^	1.72 ± 0.11^bc^	− 0.25^cde^	− 0.29^de^	− 0.17^bc^	− 0.24^de^
J2-3	2.15 ± 0.09^d^	15.8 ± 1.3^e^	1.63 ± 0.08^c^	− 0.33^e^	− 0.33^e^	− 0.21^c^	− 0.29f.
J3-1	2.81 ± 0.12^b^	19.3 ± 1.0^b^	1.81 ± 0.10^b^	− 0.13^b^	− 0.18^b^	− 0.12^b^	− 0.14^b^
J3-2	2.61 ± 0.09^bc^	18.2 ± 0.9^bcd^	1.73 ± 0.12^bc^	− 0.19^bc^	− 0.23^bcd^	− 0.16^bc^	− 0.19^ cd^
J3-3	2.37 ± 0.13^ cd^	17.3 ± 1.1^cde^	1.73 ± 0.08^bc^	− 0.26^cde^	− 0.26^cde^	− 0.16^bc^	− 0.23^de^

Data are the mean ± standard deviation of three replicates. Different lowercase letters in each column represent significant differences at the 0.05 level.

*CK* blank control, *RI* response index, *SE* synergistic allelopathy effect index.

Overall, in the seedling fresh weight growth test, except in J3-2 and J3-3, the phytotoxic effect was observed to increase gradually as the concentration of the same-year extract increased, with the phytotoxic inhibitory effect showing the trend 60 g/L > 40 g/L > 20 g/L. Tiantao et al.^31^ found that *Datura* has strong autotoxicity that increases with the concentration and may lead to issues with continuous cropping, which is consistent with the results of this study. Under the same concentration conditions, the allelopathic inhibition effect of sugarcane root extract showed an overall trend of increasing and then decreasing as the ratoon age increased, with allelopathic inhibition decreasing in the following order: J2 > J1 > J3. Studies have shown that the composition and concentration of allelochemicals are the main factors affecting allelopathy^32^. The different compositions of the allelochemicals released by decomposed sugarcane roots from ratoons of different age may have been the main reason for the observed differences in allelopathy.

### SI of receptors for different extracts and differences in receptor sensitivity

**Figure 1:**
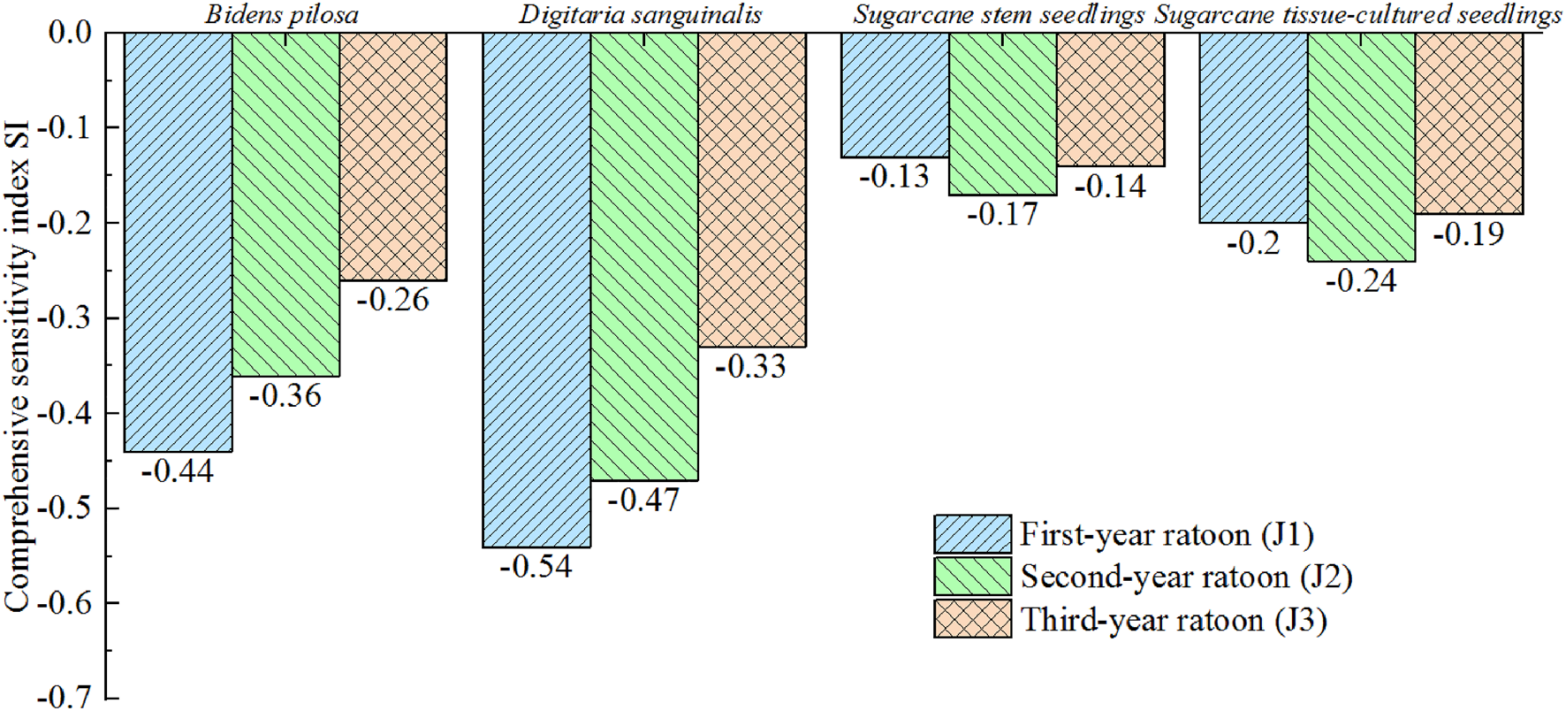
Allelopathic sensitivity indices for the four studied receptors in response to different extracts.

The SI of the receptors can be used to measure the overall strength of a donor’s comprehensive allelopathy and evaluate the sensitivity of the receptors. Figure 1 indicates that the allelopathic inhibition strength on *B. pilosa and D. sanguinalis* seeds was strongest for J1 and weakest for J3. The sensitivity of these two weed species to allelopathy from different-year root extracts decreased in the following order: J1 > J2 > J3. The allelopathic inhibition of sugarcane stem seedlings was strongest for J2 and weakest for J1, with the sensitivity of the seedlings to allelopathy of different-year root extracts decreasing in the following order: J2 > J3 > J1. The allelopathic inhibition of sugarcane tissue-cultured seedlings was strongest for J2 and weakest for J3, with the sensitivity of the seedlings to allelopathy from different-year root extracts decreasing in the following order: J2 > J1 > J3. These results indicated that the root exudate extracts from different years had varying effects on the germination and seedling growth of each receptor, with J1 exhibiting the strongest inhibitory effect on *B. pilosa and D. sanguinalis* germination and J2 exhibiting the strongest inhibitory effect on germination of sugarcane stem seedlings and tissue-cultured seedlings.

The greater the comprehensive SI of a substance to other plant species, the greater its impact on those other plants, and may also suggest stronger allelopathy. If the receptor plants are considered as a whole, the allelopathy of the donor substance can be reflected on the whole; thus, the overall allelopathy of different donor treatments on a system can be reflected by the comprehensive SI of the system as a whole. As shown in Table 5, the comprehensive SIs of the system treated with J1, J2, and J3 with root extracts from different years were − 1.32, − 1.24, and − 0.92, respectively. Overall, the allelopathy of root extracts from different years of sugarcane decreased in the following order: J1 > J2 > J3. Similarly, the comprehensive SI of each receptor indicated sensitivities of *B. pilosa, D. sanguinalis*, sugarcane stem seedlings, and sugarcane tissue-cultured seedlings of − 1.07, − 1.34, − 0.44, and − 0.63, respectively, for seed germination and seedling growth, indicating that the sensitivity of different annuals also varied in response to different root extracts. The sensitivity of the companion weeds *B. pilosa* and *D. sanguinalis* was stronger than that of sugarcane stem seedlings and sugarcane tissue-cultured seedlings. Lihua et al.^25^ found that water extracts from *A. selengensis* litter had varying allelopathic potentials on the germination of different species such as ryegrass, alfalfa, and red clover, indicating that different receptor plants have differing sensitivities to allelochemicals, which is consistent with the results of this study, with different species showing different response mechanisms. Particularly, *B. pilosa and D. sanguinalis* seeds are characterised by their fragility during germination as a result of the limited nutrient and energy provisions available for embryonic growth and development, and are more susceptible to the influence of external chemical substances as a result. In contrast, sugarcane stem seedlings and tissue-cultured seedlings possessed greater robustness, displaying a stronger ability to resist phytotoxic stressors in the environment.

**Table 5:** Allelopathic sensitivity indices of the four studied receptors according to different extracts.

Donor	Treatment component	Allelopathic synthesis effect SE				Comprehensive SI
		*Bidens pilosa*	*Digitaria sanguinalis*	Sugarcane stem seedlings	Sugarcane tissue-cultured seedlings	
J1	J1-1	− 0.26	− 0.46	− 0.10	− 0.16	− 1.32
	J1-2	− 0.47	− 0.55	− 0.13	− 0.20	
	J1-3	− 0.60	− 0.62	− 0.16	− 0.25	
J2	J2-1	− 0.16	− 0.37	− 0.14	− 0.18	− 1.24
	J2-2	− 0.27	− 0.47	− 0.16	− 0.24	
	J2-3	− 0.35	− 0.57	− 0.20	− 0.29	
J3	J3-1	− 0.16	− 0.17	− 0.11	− 0.14	− 0.92
	J3-2	− 0.42	− 0.33	− 0.14	− 0.19	
	J3-3	− 0.51	− 0.49	− 0.17	− 0.23	
Comprehensive SI		− 1.07	− 1.34	− 0.44	− 0.63	

*SE* synergistic allelopathy effect index, *SI* sensitivity index.

### Effects of different extract components on germination and seedling growth of sugarcane stem

**Figure 2:**
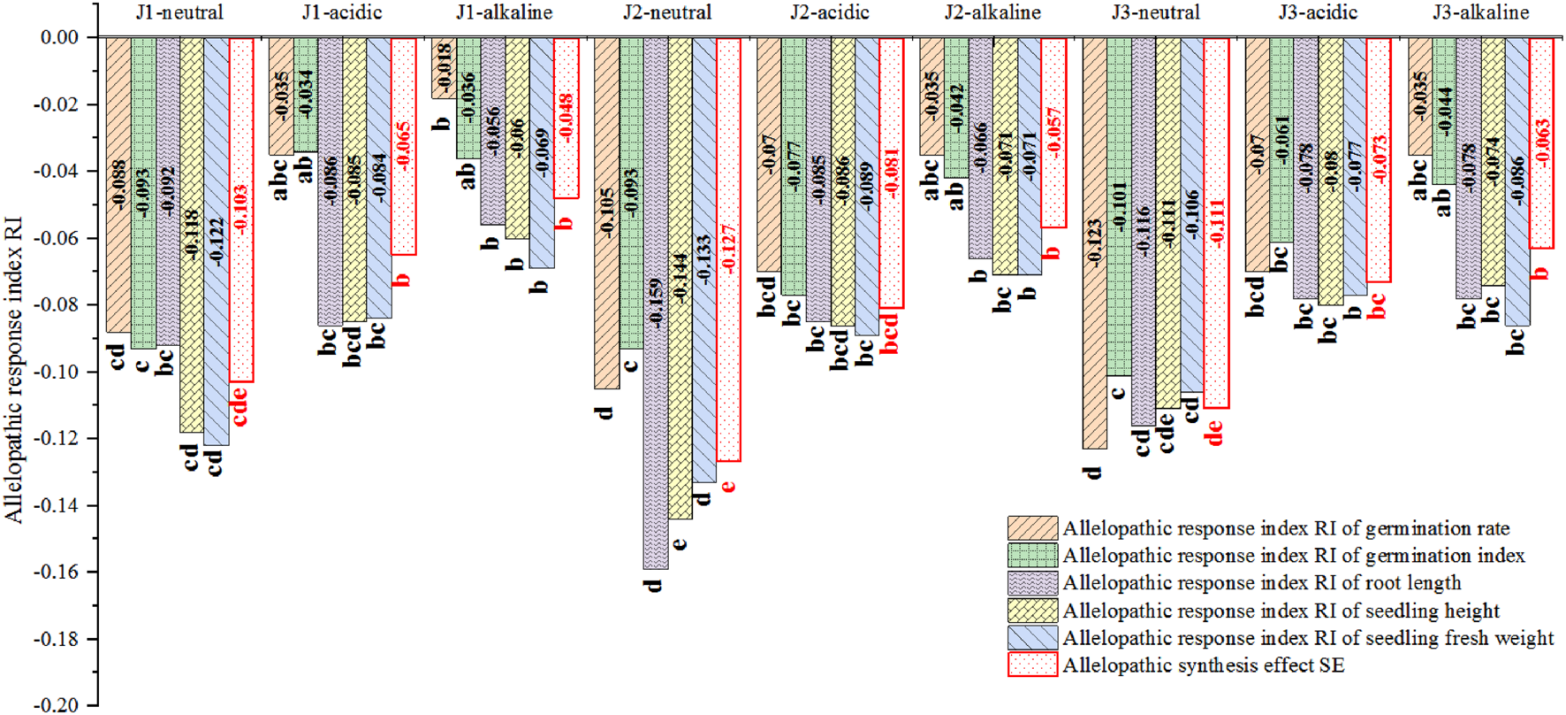
Evaluation of the allelopathy of different extract components from sugarcane ratoons of different ages on the germination of sugarcane stems and seedling growth.

The neutral, acidic, and alkaline components of extract from the root system of sugarcane plants of different ages were all observed to reduce the germination and seedling growth indicators of sugarcane stems. Specifically, the GR, GI, and seedling growth indicators were significantly lower for the J1-neutral, J2-neutral, J2-acidic, J3-neutral, and J3-acidic component treatments than for the CK (*P* < 0.05). This is consistent with the conclusions reached by Wenhong et al.^33^, who considered that the neutral, acidic, and alkaline components of *Artemisia sacrorum* water extract inhibited germination of *Achnatherum splendens* seeds. According to the comprehensive allelopathy as depicted in Fig. 2, the allelopathic inhibitory effects decreased from strongest to weakest in the following order: J2-neutral > J3-neutral > J1-neutral > J2-acidic > J3-acidic > J1-acidic > J3-alkaline > J2-alkaline > J1-alkaline. The J2-neutral treatment had the strongest comprehensive allelopathic inhibitory effect (− 0.127), and the J1-alkaline treatment had the weakest effect (− 0.048). The comprehensive inhibitory effects of all treatments in this experiment were significantly stronger than those of the CK (*P* < 0.05), with inhibitory effects for components of the perennial root system extract decreasing in the following order: J2 > J3 > J1. This result differed from the results of the comprehensive chemical sensitivity of sugarcane stem seedlings to the whole extract of the perennial root system from different years, which decreased in the following order: J2 > J1 > J3. The reason may have been interactions between different chemical components that may promote or cancel each other out^34^. The neutral fraction of the same-year perennial root extract showed a stronger allelopathic inhibition effect than other fractions, and the alkaline fraction had the weakest effect. Fenglan et al.^35^ found that the allelopathy of three fractions isolated from *Polygonum paleaceum* leaves decreased in the following order: neutral fraction > acidic fraction > alkaline fraction. Similarly, Huiyong et al.^36^ reported that the inhibitory effect of tobacco root exudates on seedling root activity decreased in the following order: neutral fraction > acidic fraction > alkaline fraction, which is consistent with the findings of this study. Wenhong et al.^33^ found that the inhibitory effect of *A. sacrorum* water extract on sand rice seed germination was stronger in the acid fraction than in the alkaline and neutral fractions. This result differs from the findings of our study. The southwestern region is the main sugarcane-producing area in China, where soil is mostly acidic or slightly acidic. Natural decomposition of root systems is likely to release more acidic and neutral components of allelochemicals into the environment. Additionally, the acidic environment formed by rainfall leaching, root secretion accumulation, soil biochemical reactions, and microbial activities can also affect the release of allelochemicals into the environment, thereby affecting the germination of sugarcane stem seeds and seedlings growth and leading to autotoxic inhibition of perennial sugarcane growth.

### Main allelochemicals and their differences of different root extract components

**Figure 3:**
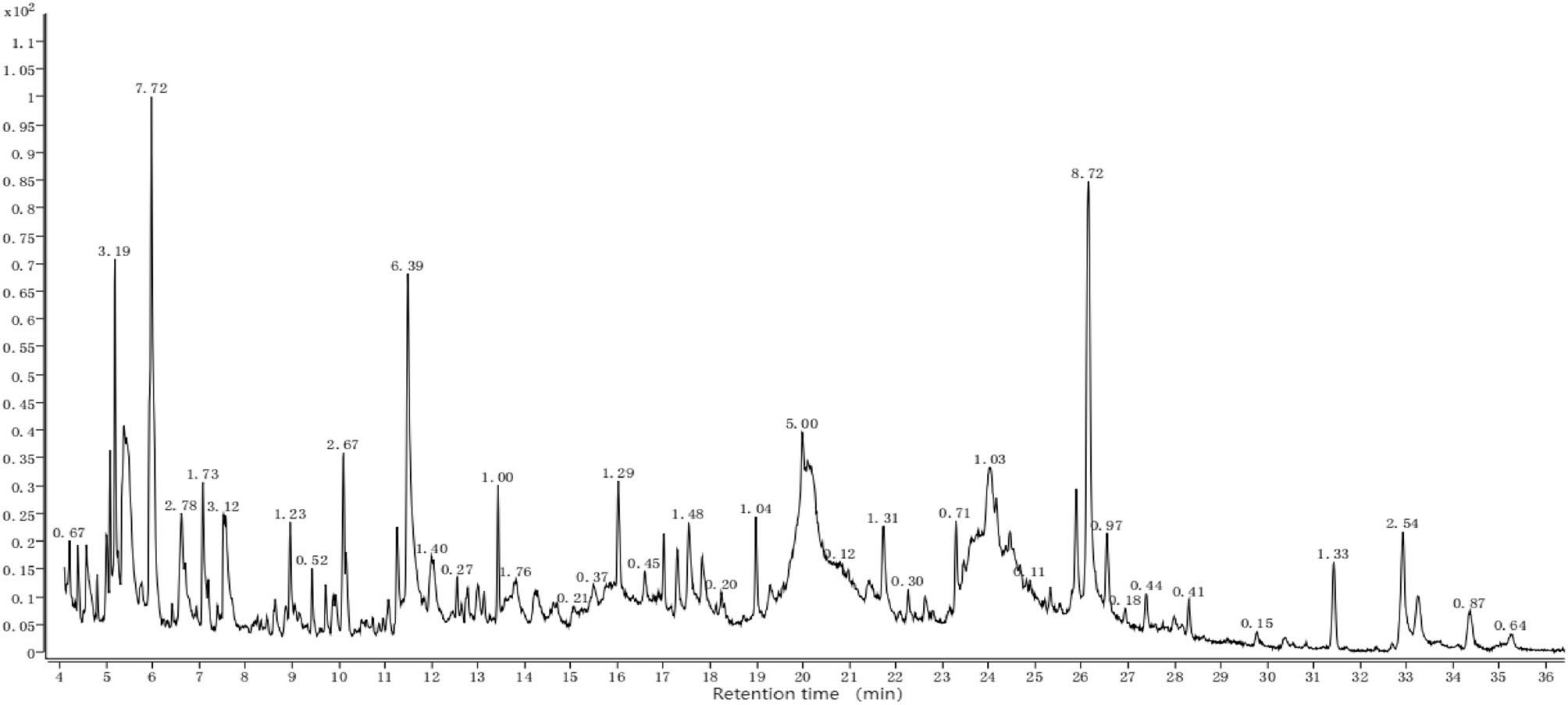
Gas chromatography–mass spectrometry (GC–MS) chromatogram of the root-extract of first-year sugarcane ratoons (J1-3).

**Figure 4:**
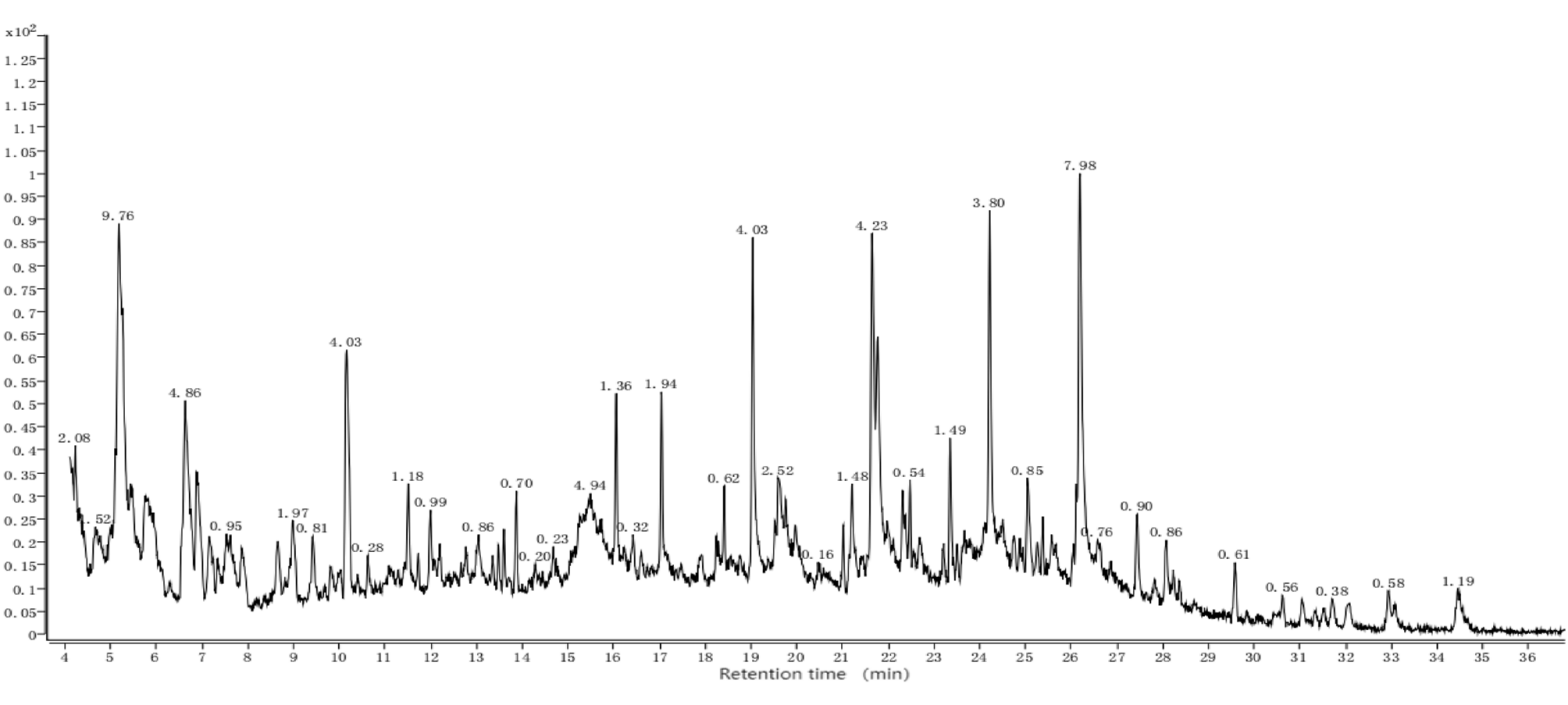
GC–MS chromatogram of the root-extract of second-year sugarcane ratoons (J2-3).

**Figure 5:**
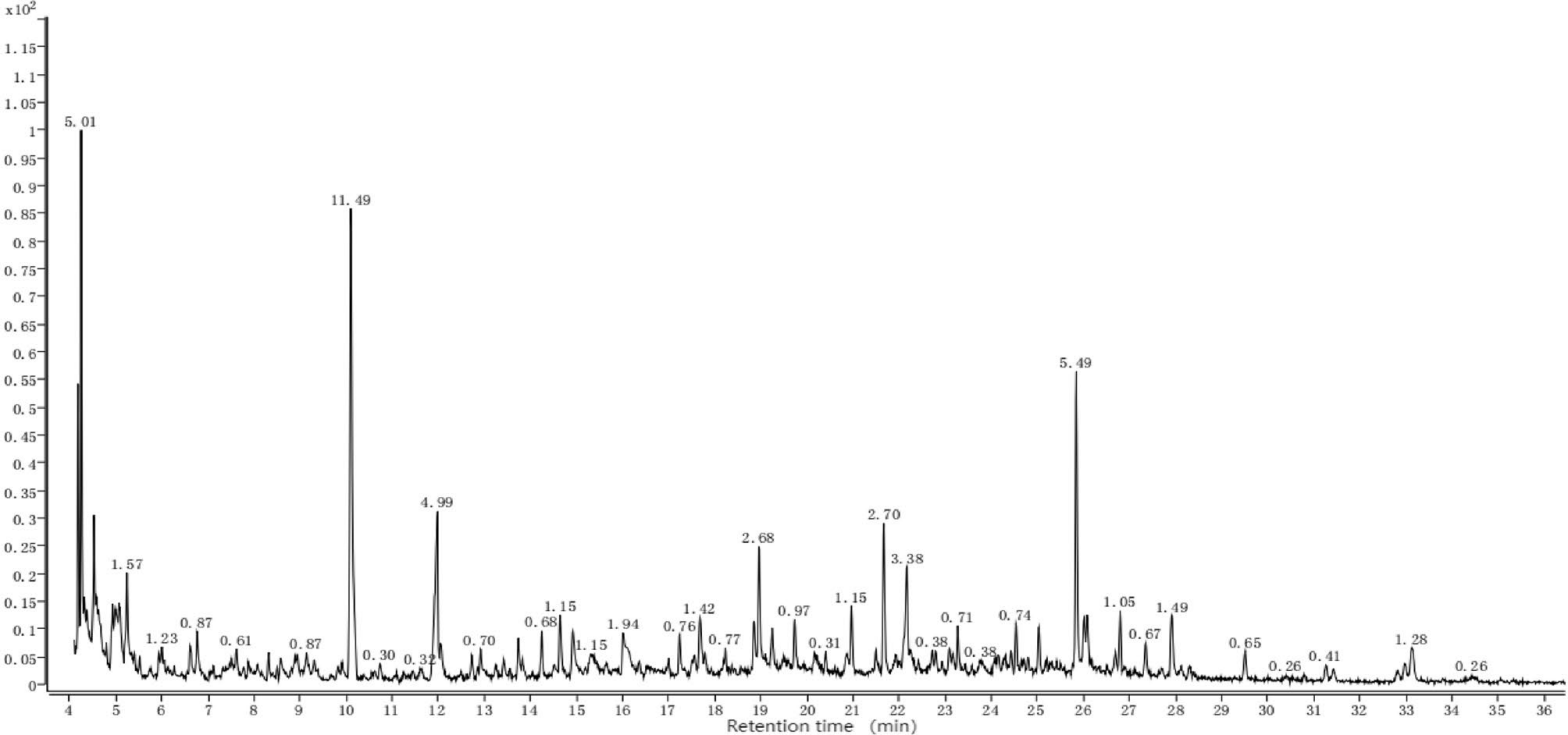
GC–MS chromatogram of the root-extract of third-year sugarcane ratoons (J3-3).

It is worth noting that our research group has conducted GC–MS analysis on the strong overall allelopathic effects from perennial sugarcane root extract of different years. Figures 3, 4, 5, Table 6 indicates that compounds in the J1-3, J2-3, and J3-3 extracts primarily comprise acids, esters, phenols, and alcohols. These compounds showed a continuous and balanced release during the decomposition of the sugarcane ratoons over the years. Acids, esters and phenols are the three component types, with more compounds detected and higher relative contents in sugarcane ratoons of different years, which could be a main reason for the allelopathic autotoxicity of sugarcane ratoons. The allelopathic effects and patterns displayed by sugarcane ratoons of different years on the receptors appeared to be related to the compounds and levels of allelochemicals in the root extracts. This finding emphasizes that the status of rhizosphere ecological factors shaped by old sugarcane ratoons of different years affects the growth of rhizospheres and plants.

**Table 6:** Allelochemicals in the root extracts of sugarcane ratoons of different ages.

Retention time (min)	Chemical compound	Relative content(%)		
		J1-3	J2-3	J3-3
4.253	3-Methoxy-2-methyl-Phenol	–	2.08	5.01
4.317	2-Chloro-3-hydroxy-butyric acid	–	–	0.71
4.394	4-(Methoxymethyl)phenol	0.67	–	–
4.585	O-xylene	–	1.52	2.10
5.183	Isopropanol	3.19	–	1.57
5.383	1,3-Dihydroxyacetone	6.42	9.76	2.07
5.973	4-Methyl-2-hexanol	7.72	1.16	1.23
6.634	Ethyl butanoate	2.78	4.86	0.87
7.160	3-Nitropropanoic acid	1.73	0.85	–
7.382	Isosorbide dinitrate	–	0.28	–
7.667	2,6-Dimethylpyridin-4-amine	3.12	0.95	0.61
9.060	(2S)-3-(Acetylsulfanyl)-2-methylpropanoic acid	1.23	1.97	0.87
9.419	Furaneol	0.52	0.81	–
10.109	Undecane	2.67	4.03	11.49
11.267	N-Methyl-N-nitroso-2-propanamine	1.09	–	–
11.497	Spermine tetrahydrochloride	6.39	1.18	–
11.980	Benzoic acid	1.13	–	4.22
12.441	4,5-Diamino-5-oxopentanoic acid	0.25	–	–
13.006	2-Dodecenoic acid	–	0.86	0.70
13.454	Isosorbide	1.00	0.52	–
13.856	3-Hydroxydodecanoic acid	1.76	0.70	0.73
14.250	Hexyl benzeneacetate	–	–	0.68
14.645	DL-mevalonolactone	–	0.23	1.15
14.922	Hydroquinone	–	–	1.03
15.522	Erythritol	–	4.94	–
16.057	3-Butanolal	1.29	1.36	1.94
17.043	Syringol	–	1.94	–
17.733	3-Hydroxybutyric acid	1.48	–	1.42
18.966	3,4-Dimethoxyphenol	1.04	4.03	2.68
19.652	Pterin-6-carboxylic acid	–	2.52	0.97
19.940	Dihydroeugenol	5.00	4.03	
20.970	3,5-Di-tert-butylphenol	–	–	1.15
21.240	N-butanoyl-DL-homoserine lactone	–	1.48	–
21.672	2,6-Dimethoxyhydroquinone	1.31	4.23	2.70
22.271	3-Hydroxy-4-methoxybenzoic acid	0.30	1.17	3.38
23.378	3,4,5-Trimethoxyphenol	0.71	1.49	0.71
24.219	Hydroconiferyl Alcohol	1.03	3.80	–
24.542	Adipic acid divinyl ester	1.15	1.12	0.74
25.059	2-Hexyl-1,1-bicyclopropane-2-octanoic acid methyl ester	–	0.85	0.64
26.150	Dihydroferulic acid	8.72	7.98	5.49
26.664	1-(4-methoxyphenyl)-1,4-butanediol	0.97	0.76	1.05
27.396	2-Amino-3-(5-nitro-3H-imidazol-4-yl)propanoic acid	0.44	0.90	0.67
27.919	2,6-Dimethyl-4-nitrophenol	–	0.86	1.49
28.314	2-(5-hydroxy-1,1,5-trimethylhexyl)-3-methylcyclopropenyl, methyl ketone	0.41	–	–
29.613	Diisobutyl phthalate	–	0.61	0.65
31.435	Methyl palmitate	1.33	0.25	0.41
33.086	Palmitic acid	2.54	0.58	1.28
33.242	(4-Hydroxy-3,5-dimethoxy-phenyl)-acetic acid methyl ester	0.92	–	–
34.428	Trans-sinapyl alcohol	0.87	1.19	–

## Conclusion

Different concentrations of root extract from perennial sugarcane in different years significantly inhibited the comprehensive allelopathy on the four studied receptors: the companion weed *B. pilosa, D. sanguinalis,* sugarcane stem seedlings, and sugarcane tissue-cultured seedlings (*P* < 0.05). Maximum comprehensive allelopathies of − 0.60, − 0.62, − 0.20, and − 0.29 were obtained, respectively. The allelopathy of the four receptors increased gradually as the concentration of the same-year root extract increased, with the allelopathic inhibition effect being greater for the 60 g/L treatment than for the 40 and 20 g/L treatments. *B. pilosa* and *D. sanguinalis* showed greater allelopathic inhibition in response to the first-year root extract than the second- and third-year extracts; whereas, the stem cuttings and tissue culture seedlings of sugarcane showed greater allelopathic inhibition in response to the second-year root extract than in to the first- and third-year extracts. The neutral, acidic, and alkaline components of the root extracts of perennial sugarcane from different years all exhibited significantly stronger comprehensive inhibitory effects on the stem germination of sugarcane than that by the CK (*P* < 0.05). Among the extracts with the same components, the inhibitory effects also varied, with the strongest response observed in the second-year extract, followed by the third- and first-year extracts. Within the same year, the inhibitory effects of the extracts with different components decreased in the following order: neutral component > acidic component > alkaline component. The results of this study suggested that the allelopathy and patterns by which perennial sugarcane root extract affects receptors vary in different years, acids, esters and phenols could be a main reason for the allelopathic autotoxicity of sugarcane ratoons and depending on the type and content of allelochemicals in the root extract. The differences in the allelopathy of the root extract components also indicated that allelopathy was influenced by other environmental factors, and that ecological factors in the rhizosphere that result from the presence of old perennial sugarcane root from different years affected plant growth. Our previous research^37^ demonstrated that the root extract of perennial sugarcane may induce allelopathy in three ways. First, it can affect the accumulation levels of osmotic regulatory substances in sugarcane stem seedlings. Second, it can cause peroxidation damage to the cell membrane of the sugarcane stem seedlings. Third, it can alter the activity of various enzymes in the rhizosphere environment. The perennial sugarcane root system is the primary “shaper” and “participant” in the rhizosphere ecological environment. The rhizosphere ecological factors of sugarcane perennial roots are an essential component of plant‒soil feedback functions. While exhibiting allelopathy, the old roots of perennial sugarcane also showed autotoxicity, which may be a crucial factor contributing to the decline of perennial sugarcane root health.

## Data Availability

The datasets used or analyzed in the current study are available from the corresponding author upon a reasonable request.
